# Well-TEMP-seq as a microwell-based strategy for massively parallel profiling of single-cell temporal RNA dynamics

**DOI:** 10.1038/s41467-023-36902-5

**Published:** 2023-03-07

**Authors:** Shichao Lin, Kun Yin, Yingkun Zhang, Fanghe Lin, Xiaoyong Chen, Xi Zeng, Xiaoxu Guo, Huimin Zhang, Jia Song, Chaoyong Yang

**Affiliations:** 1 State Key Laboratory of Physical Chemistry of Solid Surfaces, MOE Key Laboratory of Spectrochemical Analysis & Instrumentation, Key Laboratory for Chemical Biology of Fujian Province, Innovation Laboratory for Sciences and Technologies of Energy Materials of Fujian Province, Department of Chemical Biology, College of Chemistry and Chemical Engineering, Xiamen University, Xiamen, 361005 China; 2 Institute of Molecular Medicine, State Key Laboratory of Oncogenes and Related Genes, Renji Hospital, School of Medicine, Shanghai Jiao Tong University, Shanghai, 200120 China; 3 Department of Neurosurgery, Neurosurgery Research Institute, the First Affiliated Hospital of Fujian Medical University, Fuzhou, 350000 China

**Keywords:** RNA sequencing, RNA, Transcriptomics, Microfluidics

## Abstract

Single-cell RNA sequencing (scRNA-seq) reveals the transcriptional heterogeneity of cells, but the static snapshots fail to reveal the time-resolved dynamics of transcription. Herein, we develop Well-TEMP-seq, a high-throughput, cost-effective, accurate, and efficient method for massively parallel profiling the temporal dynamics of single-cell gene expression. Well-TEMP-seq combines metabolic RNA labeling with scRNA-seq method Well-paired-seq to distinguish newly transcribed RNAs marked by T-to-C substitutions from pre-existing RNAs in each of thousands of single cells. The Well-paired-seq chip ensures a high single cell/barcoded bead pairing rate (~80%) and the improved alkylation chemistry on beads greatly alleviates chemical conversion-induced cell loss (~67.5% recovery). We further apply Well-TEMP-seq to profile the transcriptional dynamics of colorectal cancer cells exposed to 5-AZA-CdR, a DNA-demethylating drug. Well-TEMP-seq unbiasedly captures the RNA dynamics and outperforms the splicing-based RNA velocity method. We anticipate that Well-TEMP-seq will be broadly applicable to unveil the dynamics of single-cell gene expression in diverse biological processes.

## Abstract (web summary)

Gene expression of cells is a heterogeneous and dynamic program involved in various biological processes. Here, authors develop Well-TEMPseq, a high-throughput, cost-effective, and accurate method for massively parallel profiling of the temporal dynamics of single-cell gene expression.

## Introduction

Gene expression of cells is a heterogeneous and dynamic program that changes over time in various biological processes such as cellular differentiation, embryonic development, disease progression, and responses to external stimuli^1–6^. ScRNA-seq has been widely applied to reveal the heterogeneity of cells and discover novel cell types^7,8^. However, most scRNA-seq methods capture static snapshots of single-cell gene expression and fail to temporally resolve the RNA dynamics^8^. Pseudotime-based methods analyze scRNA-seq data of cells with different states, order cells by their transcriptome similarity, and infer a continuous trajectory to reveal the biological progression^9,10^. Nevertheless, pseudotime ordering does not provide the true and precise dynamics of gene expression for resolving the directionality of complex biological processes^11^. The recently proposed concept of “RNA velocity” tries to describe the states of cells based on the time derivatives of unspliced and spliced RNA abundance and predict the future states by extrapolation^12,13^. But it is challenging to predict the continuous evolution from historical states to future states without the knowledge of nascent RNA and pre-existing RNA abundance.

Metabolic RNA labeling is broadly used to label newly transcribed RNAs with exogenous nucleoside analogs and distinguish new RNAs from pre-existing RNAs, yielding critical insights into the RNA dynamics^14,15^. Recent advancement in scRNA-seq technology has demonstrated the feasibility of combining metabolic RNA labeling with scRNA-seq to profile both new and old transcriptomes at the single-cell level^16–20^. For example, NASC-seq, scSLAM-seq, and scEU-seq integrate plate-based scRNA-seq with metabolic RNA labeling (4-thiouridine, 4sU or 5-ethynyl uridine, 5-EU) to identify new RNAs in each of single cells^16–18^. However, these methods are low-throughput, costly, time-consuming, and labor-intensive. Recently developed methods such as sci-fate and scNT-seq adopt combinational indexing or droplet-based microbead barcoding to enhance the throughput and reduce the cost. Nevertheless, combinational indexing in sci-fate requires in situ 4sU chemical conversion in fixed cells and multiple centrifugation steps, leading to a high cell loss rate (>95%) and potential RNA degradation. Even though scNT-seq uses barcoded beads to capture RNAs of single cells and avoid in situ chemical conversion-induced cell loss, cell-free RNAs in the droplets cannot be washed away and the Poisson distribution-dependent droplet barcoding results in a low single cell/barcoded bead pairing efficiency (<1%)^19,20^. To date, it remains urgent but challenging to develop a high-throughput, low-cost, and accurate method with high barcoding efficiency and low cell loss rate for capturing the temporal dynamics of transcription in single cells.

To tackle these constraints, we develop Well-TEMP-seq, a high-throughput and cost-effective method that combines metabolic RNA labeling by 4sU with our size-exclusion and quasi-static microwell-based Well-paired-seq^21^ to quantify new and old transcriptomes in each of thousands of single cells without complicated equipment. Well-TEMP-seq enables highly efficient Poisson distribution-independent single cell/bead pairing, cell-free RNA removal, and unique molecular identifiers (UMIs)-based counting to accurately profile newly synthesized RNAs and pre-existing RNAs. We demonstrate that Well-TEMP-seq characterizes the gene expression dynamics of colorectal cancer cells treated with a low dose of anti-tumor drug (5-AZA-CdR), where 5-AZA-CdR-induced global DNA demethylation results in the re-activation of tumor suppressor genes and the repression of oncogenes. Well-TEMP-seq also reveals the 5-AZA-CdR-induced early-stage activation (e.g., in the first three days) of interferon-responsive transcription factor by viral mimicry. We anticipate that Well-TEMP-seq will be broadly applicable to other biological systems to characterize the temporal dynamics of gene expression at the single-cell level.

## Results

### Working principle of Well-TEMP-seq

**Fig. 1:**
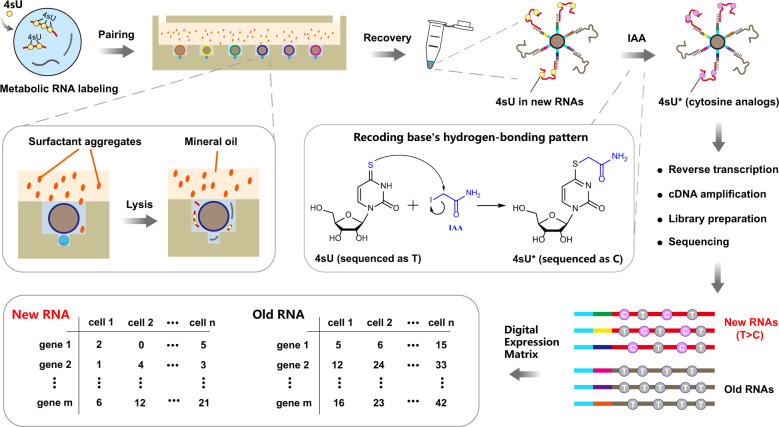
Overview and workflow of Well-TEMP-seq. Cells are first incubated with 4sU to label newly transcribed RNAs and are then loaded into Well-paired-seq chip for single cell/bead pairing. After cell lysis by surfactant aggregates (sakosyl), RNAs are captured by DNA-barcoded beads and subjected to IAA (iodoacetamide) treatment to transform 4sU to a cytosine analog (4sU*), which induces T-to-C substitutions at 4sU-labeled sites. RNAs on the beads are reverse transcribed and the resulting cDNAs are amplified for library preparation and sequencing. Reads with T-to-C substitution(s) are identified as new RNAs while others are old RNAs. Therefore, each cell is associated with two digital expression matrices (new RNA and old RNA).

Well-TEMP-seq is built on our recently developed size-exclusion and locally quasi-static hydrodynamic microwell-based single-cell RNA sequencing platform (Well-paired-seq) with high throughput, low cost, and high efficiency. The Well-TEMP-seq method relies on the following key steps (Fig. 1): (1) Cells are incubated with 4sU, the biocompatible thymidine analog, to label newly transcribed RNAs. (2) Cells and barcoded microbeads with oligo(dT) primers are successively loaded into the microwells to achieve single-cell/bead pairing. Cell-free RNAs can be removed by washing before loading microbeads. (3) Cells are lysed by sakosyl released from surfactant aggregates in the covering oil layer and RNAs with poly(A) tails are captured by the microbeads with oligo(dT) primers. (4) Microbeads are pooled and subjected to a one-pot chemical reaction with iodoacetamide (IAA) to recode base’s hydrogen-bonding pattern and transform 4sU to a cytosine analog by nucleophilic substitution, which results in T-to-C substitutions at 4sU labeled sites in the following reverse transcription. (5) RNAs on the microbeads are reverse transcribed and the resulting cDNAs are amplified by PCR and tagmented by Tn5 transposase for library preparation. (6) After sequencing, reads with T-to-C substitution(s) are identified as newly synthesized transcripts and calculated by a unique molecular identifier (UMI)-based model to infer the fraction of new RNAs of each gene in each cell. Therefore, each cell is associated with two digital expression matrices (new and old RNA). It is noteworthy to mention that Well-TEMP-seq can handle up to 8 parallel samples in one chip, which is beneficial for reducing batch effects^21^. Moreover, all the operations of loading cells and microbeads are accomplished with an optical microscope and a pipette, which makes this technique readily affordable and reproducible in other labs.

### Validation of the Well-TEMP-seq performance

**Fig. 2:**
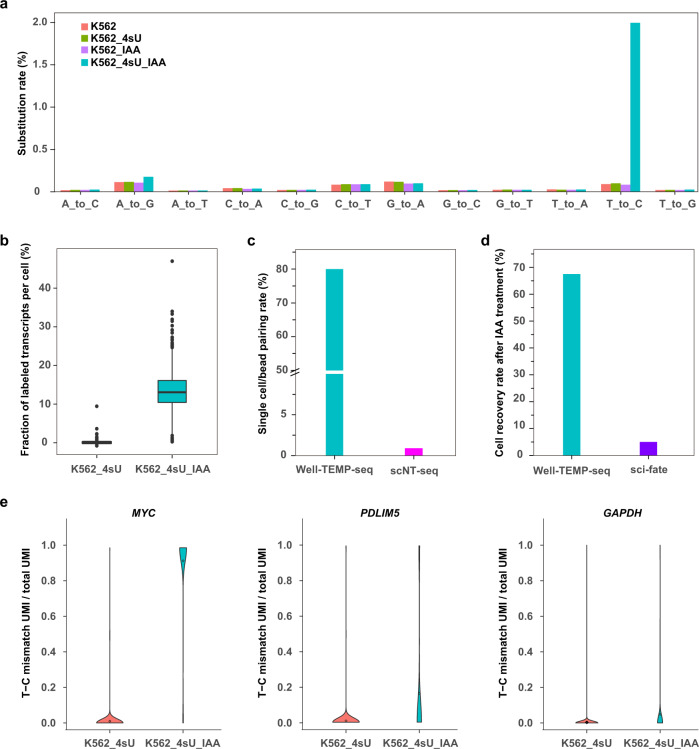
Validation of the Well-TEMP-seq performance. **a** Bar plot of nucleotide substitution rates in K562 cells (K562), 4sU-labeled K562 cells (K562_4sU), IAA-treated K562 cells (K562_IAA), and 4sU-labeled and IAA-treated K562 cells (K562_4sU_IAA). **b** Box plot of the fraction of labeled transcripts per cell in 4sU-labeled K562 cells and 4sU-labeled and IAA-treated K562 cells. *n* = 2000 cells for each group. Boxplots include centerline, median; box limits, upper and lower quartiles; and whiskers are highest and lowest values no >1.5× interquartile range. **c** Single-cell/barcoded bead pairing rate in Well-TEMP-seq and scNT-seq. The pairing rate in scNT-seq was calculated by Poisson distribution. **d** Cell recovery rate after IAA chemical treatment in Well-TEMP-seq and sci-fate. **e** Violin plots showing the fraction of T-C mismatch UMIs per cell of different genes in 4sU-labeled K562 cells and 4sU-labeled and IAA-treated K562 cells. Left, high turnover gene (*MYC*); middle, median turnover gene (*PDLIM5*); right, low turnover gene (*GAPDH*). Source data are provided as a Source Data file.

We first investigated the labeling efficiency and chemical conversion ability of Well-TEMP-seq. We modified the 4sU-IAA reaction condition by reducing the amount of dimethylsulfoxide (DMSO) and lowering the reaction temperature, making it compatible with on-bead chemical conversion. As shown in Fig. 2a and Supplementary Fig. 1a, the cells labeled with 4sU (200 μM, 2 h) and chemically treated with IAA exhibited high T-to-C substitution rates. Other cells without metabolic labeling or IAA treatment showed negligible substitution rates, indicating a high signal-to-noise ratio for Well-TEMP-seq. After 2 h labeling of K562 cells with 4sU, the median fraction of labeled reads in each cell was about 15% (Fig. 2b), which was close to that of previous reports^12,19^. The newly transcribed RNAs were marked by a higher portion of 4sU labeled reads mapping to introns versus exons, which was consistent with previous works that the intronic reads were more likely from newly synthesized RNAs (Supplementary Fig. 1b)^12,19^. It was noted that chemical treatment by IAA would lead to partial RNA degradation and negatively affect the library complexity (Supplementary Fig. 1c,d, Supplementary Fig. 2, and Supplementary Data 1). However, Well-TEMP-seq still retained high gene detection ability (>3000 genes at 50,000 reads per cell, Supplementary Fig. 1d), which was enough for downstream applications of characterizing the temporal dynamics of single-cell gene expression.

Besides, the size-exclusion and locally quasi-static hydrodynamic microwell allowed highly efficient single cell/barcoded bead pairing with a pairing rate of ~80%, which was significantly higher than that of Drop-seq-based scNT-seq (Fig. 2c). Poisson distribution-dependent pairing in scNT-seq resulted in the low pairing rate (<1%). Moreover, IAA chemical treatment was performed on beads in Well-TEMP-seq, which successfully minimized the cell loss (~67.5% recovery, Fig. 2d). In contrast, in situ chemical conversion in sci-fate required multiple centrifugation steps and led to severe cell loss (<5% recovery). Therefore, the workflow of Well-TEMP-seq enabled highly efficient single-cell/bead pairing and cell recovery.

To further examine the accuracy of Well-TEMP-seq in identifying newly transcribed RNA, we analyzed genes encoding mRNAs with different turnover (high, median, and low) as determined by previous reports^22^. The mRNAs with high turnover should have a larger fraction of newly transcribed RNAs, as indicated by a larger fraction of reads with T-to-C substitution(s) in Well-TEMP-seq data. Indeed, we did observe different genes with high (*MYC*), intermediate (*PDLIM5*), or low (*GAPDH*) turnover that showed high, intermediate, and low fractions of reads with T-to-C substitution(s), respectively (Fig. 2e). Global analysis of all detected genes in K562 cells and HCT116 cells further confirmed that mRNAs with high turnover have a larger fraction of newly transcribed RNAs (Supplementary Fig. 1e,f). Therefore, these results indicated that labeling and subsequent sequencing of newly transcribed RNAs in individual cells by Well-TEMP-seq are feasible.

### Characterizing transcriptome dynamics in 5-AZA-CdR response

To demonstrate the ability of Well-TEMP-seq to resolve temporal dynamics of single-cell gene expression, we applied Well-TEMP-seq to profile the transcriptome dynamics of colorectal cancer cells in response to 5-AZA-CdR, an anti-tumor DNA-demethylating agent. Decitabine (also called 5-AZA-CdR) is a FDA-approved DNA methylation-inhibiting drug^23,24^. After incorporating into DNA, 5-AZA-CdR, the cytidine analog can trap DNA methyltransferases, induce proteasomal degradation, and result in heritable global DNA demethylation^25^. However, it is still unclear whether the clinical efficacy of 5-AZA-CdR is from promoter demethylation of aberrantly methylated tumor suppressor genes (TSGs) or the consequence of 5-AZA-CdR-induced double-stranded RNA (dsRNA) expression and the resultant interferon response pathway^24^. Moreover, the early-stage (e.g., the first three days) gene expression dynamics of colorectal cancer cells treated with 5-AZA-CdR is still unknown due to the lack of sensitive methods to unravel the changes of transcription states in the short period.

**Fig. 3:**
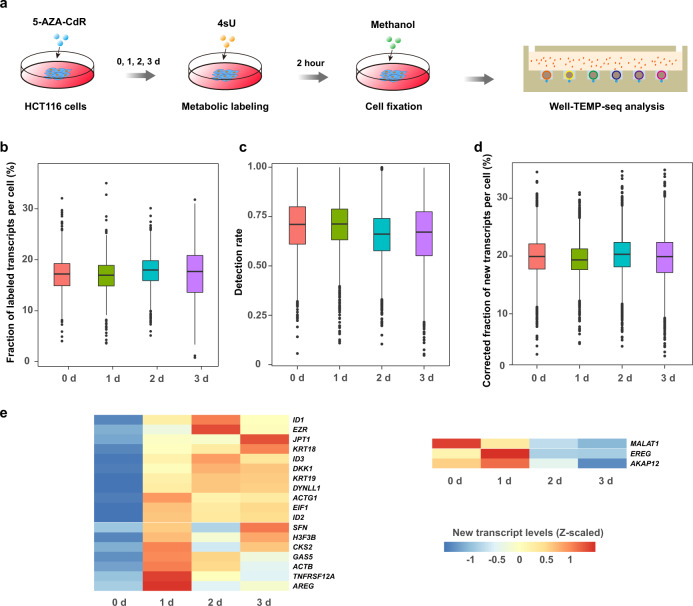
Well-TEMP-seq characterizes the transcriptome dynamics of HCT116 cells in 5-AZA-CdR response. **a** Experimental scheme of characterizing the transcriptome dynamics of 5-AZA-CdR-treated HCT116 cells with Well-TEMP-seq. Cells were treated with 300 nM 5-AZA-CdR for 0/1/2/3 d. Cells in each group were labeled with 200 μM 4sU for 2 h before fixation for Well-TEMP-seq. **b** Box plot showing the fraction of labeled transcripts per cell in different groups before correction. Here, correction is essential since current metabolic RNA labeling strategies may cause incomplete 4sU labeling of newly transcribed RNAs due to the presence of pre-existing uridine in cells and false positives arising from PCR errors and sequencing. **c** Box plot of detection rate of different groups. Calculated as the ratio between observed and corrected newly transcribed RNA levels for each cell, the detection rate is an important coefficient in the correction process and represents the labeling efficacy of newly transcribed RNAs. **d** Box plot showing the fraction of new transcripts per cell in different groups after correction. In **b**–**d**, *n* = 872 (0 d), 867 (1 d), 884 (2 d), 832 (3 d) cells and boxplots include centerline, median; box limits, upper and lower quartiles; and whiskers are highest and lowest values no >1.5× interquartile range. **e** Heat maps showing average new RNA levels (z-scaled natural log transformation of (TP10K + 1)) of significantly up-regulated genes (left) and down-regulated genes (right) in response to 5-AZA-CdR treatment. Source data are provided as a Source Data file.

As depicted in Fig. 3a, we first treated HCT116 colorectal cancer cells with low-dose (300 nM) 5-AZA-CdR for different durations (0 day, 1 day, 2 days, and 3 days) and labeled the nascent RNAs with 4sU (200 μM) for 2 h immediately preceding harvest. Harvested cells were fixed with methanol to preserve their states of each condition. Then we performed parallel Well-TEMP-seq of the 4 samples (0 day, 1 day, 2 days, and 3 days) to minimize potential batch effects. After quality filtering, we obtained paired single-cell new and old transcriptomes of about 16,000 cells (Supplementary Fig. 3 and Supplementary Data 2). Current metabolic RNA labeling strategies may cause incomplete 4sU labeling of newly transcribed RNAs due to the presence of pre-existing uridine in cells and lead to inaccurate fraction of new transcripts for each gene in each cell (Fig. 3b). When using T-to-C substitutions as a proxy for newly transcribed RNAs, false positives may arise from PCR errors and sequencing. To overcome these issues, we adapted a binomial mixture model from the recently established GRAND-SLAM statistical correction approach^26^ and optimized it for UMI-based Well-TEMP-seq datasets. The expression level of new transcript for each gene at each time point was obtained from the maximum likelihood function of the statistical model (detailed in Methods). We calculated the detection rate, α (the ratio between observed and corrected newly transcribed RNA levels in individual cells) by dividing the number of all observed labeled transcripts by the number of all estimated new transcripts for each cell (Fig. 3c). Then the corrected new RNA level of each gene in each cell was inferred by dividing the observed new RNA level by α (Fig. 3d).

After correction, we performed differential gene expression (DGE) analysis of new transcripts to identify genes in response to 5-AZA-CdR treatment, by comparing the new RNA level of each treated group with that of untreated group (i.e., 1 d vs. 0 d, 2 d vs. 0 d, 3 d vs. 0 d). Genes with an absolute fold change of new RNA expression >1.5 and an adjusted *P* value <0.05 (Bonferroni corrected) in at least one treated group were considered as differentially expressed genes. Compared with gene expression level of cells in the untreated group (0 d), 18 and 3 genes were significantly up-regulated and down-regulated, respectively (Fig. 3e). Previous studies revealed that a low-dose 5-AZA-CdR treatment after five days could induce global DNA demethylation in HCT116 cells^23,24^. Genes with hypermethylated promotors will be re-activated while genes with methylated gene bodies will be repressed upon 5-AZA-CdR treatment. We found that TSGs (e.g., *DKK1* and *GAS5*) in colorectal cancer with hypermethylated promotors were re-activated and two oncogenes (i.e., *EREG* and *MALAT1*) were down-regulated upon 5-AZA-CdR treatment. The re-activation of hypermethylated TSGs and repression of overexpressed oncogenes by 5-AZA-CdR would synergistically assert clinical anti-tumor efficacy. Due to its ability to quantify the newly synthesized RNAs, Well-TEMP-seq is more sensitive to reveal the dynamic changes of gene expression in the early stage. Therefore, our single-cell gene expression results revealed the activation of *DKK1* and *GAS5* and the suppression of *EREG* and *MALAT1* in response to the low-dose 5-AZA-CdR treatment in the first three days. It should be noted that we also observed a set of up-regulated genes that were candidate cancer driver genes. Even though the global demethylation induced by 5-AZA-CdR re-activated TSGs and suppressed oncogenes, the upregulation of potential cancer driver genes may raise concerns for potential side effects of 5-AZA-CdR treatment.

### Identification of 5-AZA-CdR-induced, time-dependent regulon activity

**Fig. 4:**
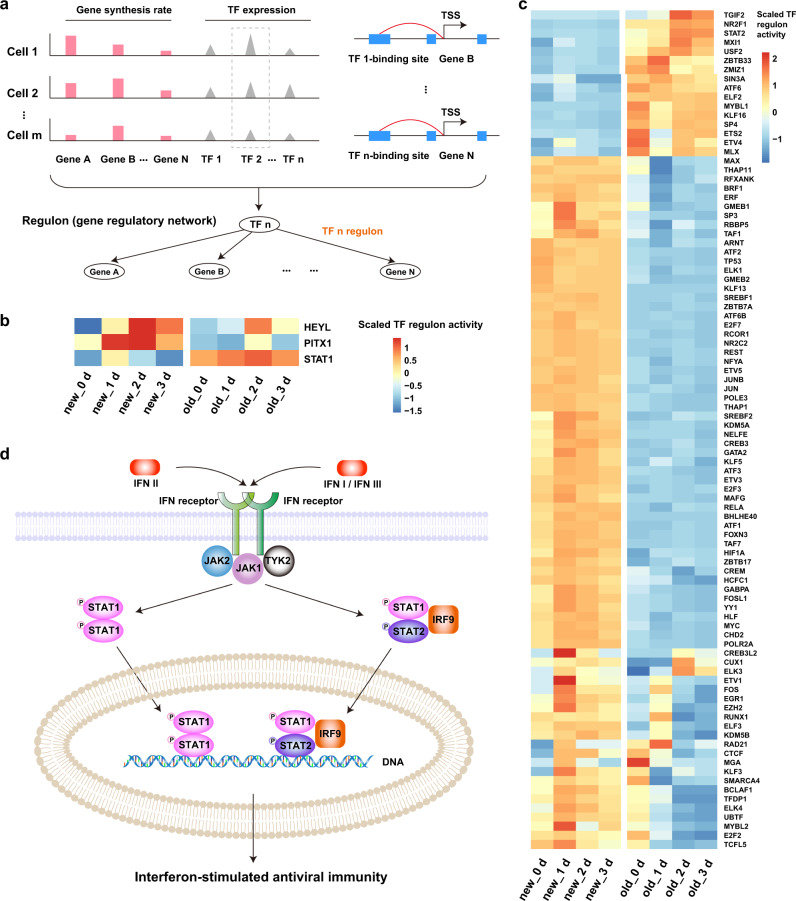
Analysis of treatment-dependent regulon activity of HCT116 cells in response to 5-AZA-CdR. **a** Schematic illustration of regulon identification by linking TFs with their regulating genes. **b** Heat map showing average regulon activity of 3 5-AZA-CdR treatment-dependent regulons of HCT116 cells, inferred from either new or old RNAs. Regulons with significantly increased or decreased activities (absolute fold change >1.5) were identified by a two-sided Wilcoxon rank-sum test (adjusted *P* value <0.05, Bonferroni corrected). **c** Clustered heat map showing the average regulon activity of 92 5-AZA-CdR treatment-independent regulons of HCT116 cells, inferred from either new or old RNAs. **d** Simplified diagram of the IFNI/II/III-associated induction and receptor signaling pathway, leading to the activation of JAK/TYK-STAT pathway and interferon-stimulated antiviral immunity. Source data are provided as a Source Data file.

Gene expression is tightly regulated by DNA-binding transcription factors (TFs). The coordinated expression of TFs and their respective sets of genes forms the complex gene regulatory networks (GRNs) (Fig. 4a). The pattern of the activities of regulons (i.e., TFs and their target genes) represents the distinct transcriptional state and even cell identity of each cell. Regulon activities of TFs can be quantified by linking cis-regulatory sequences to single-cell gene expression, as demonstrated by the recently proposed single-cell regulatory network inference and clustering (SCENIC) method^27,28^. By applying SCENIC to paired single-cell new and old transcriptomes from Well-TEMP-seq, we identified 95 co-regulated TF regulons with significant cis-regulatory motif enrichment (Figs. 4b, c). With newly transcribed RNAs, SCENIC analysis revealed three regulons (i.e., STAT1, HEYL, and PITX1) that exhibited significant changes in response to 5-AZA-CdR treatment (Fig. 4b). Although regulons exhibiting significant changes in response to 5-AZA-CdR treatment were also identified in old RNA-based SCENIC analysis and total RNA-based SCENIC analysis (Supplementary Fig. 4), we focused on the new RNA-based SCENIC results since new RNAs more faithfully revealed the 5-AZA-CdR treatment responsive regulation.

STAT1 is a signal transducer and transcription activator involved in cellular interferon response^29^. Prior reports established that low-dose 5-AZA-CdR induced RNA polymerase III-driven bidirectional transcription of normally silent repeats^30^. The resultant formation of dsRNA further activated the MDA/MAVS/IRF7 interferon responsive pathway by viral mimicry and produced interferons (IFNs) (Supplementary Fig. 5)^24^. After interferon stimulation, STAT1 is phosphorylated and activated, which further forms pSTAT1 homodimer or pSTAT1/pSTAT2/IRF9 complex to activate antiviral immunity (Fig. 4d)^31–33^. It has been revealed in previous reports that a low-dose 5-AZA-CdR treatment for more than five days induced STAT1 activation in HCT116 cells. However, it remains unknown whether STAT1 is activated in the early stage of treatment (e.g., the first three days). Our results revealed that the 5-AZA-CdR treatment enhanced STAT1 phosphorylation (Supplementary Fig. 6a), the expression of STAT1 target genes (Supplementary Fig. 6b), and the STAT1 regulon activity (Fig. 4b). These results have filled the gap and proved that STAT1 was activated in the first three days upon treatment.

HEYL is a downstream effector of Notch signaling pathway and is frequently down-regulated by promoter hypermethylation, leading to the inactivation of HEYL-associated P53-mediated apoptosis in cancer cells^34,35^. It has been revealed that HEYL inhibited tumor cell dissemination and decreased the metastasis-forming capacity of colorectal cancer and hepatocellular carcinoma^36^. Therefore, the 5-AZA-CdR treatment-dependent enhancement of HEYL regulon activity is beneficial for the anti-tumor effects.

PITX1 is a critical transcription factor involved in suppressing the tumorigenicity of multiple human cancers, including colorectal cancer^37^. The altered RAS pathway by mutated RAS overexpression has been frequently observed in colorectal tumors^38^. PITX1 plays a tumor suppressor role by downregulating the RAS pathway^37,39^. It also has been revealed that PITX1 may activate TP53 apoptosis pathway and suppress the activity of telomerase reverse transcriptase^37,40,41^. However, the low expression of PITX1 is frequently observed in tumors^37^. The treatment of 5-AZA-CdR re-activated the activity of PITX1 regulon and may restore the tumor suppressive functions of PITX1.

Thus, thanks to the high sensitivity of Well-TEMP-seq, we can provide insights into the 5-AZA-CdR induced anti-tumor response of STAT1, HEYL, and PITX1 TFs activation in the early stage of treatment (e.g., in the first three days), which has not been unveiled before. The activation of STAT1-related antiviral immunity and the restoration of tumor suppressive TF activities of HEYL and PITX1 would synergistically contribute to the anti-tumor effects of 5-AZA-CdR treatment.

### Metabolic RNA labeling-based RNA velocity analysis of 5-AZA-CdR response

The concept of “RNA velocity” has recently been proposed as the time derivative of gene expression to predict the future state (on the scale of hours) of an individual cell. RNA velocity can be inferred from scRNA-seq datasets by splicing-based model or labeling-based model to inform the temporal dynamics of gene expression in single cells. To quantify the RNA velocity of HCT116 cells exposed to low-dose 5-AZA-CdR, we adopted dynamo^42^, a recently established computational method, to analyze the datasets from Well-TEMP-seq.

**Fig. 5:**
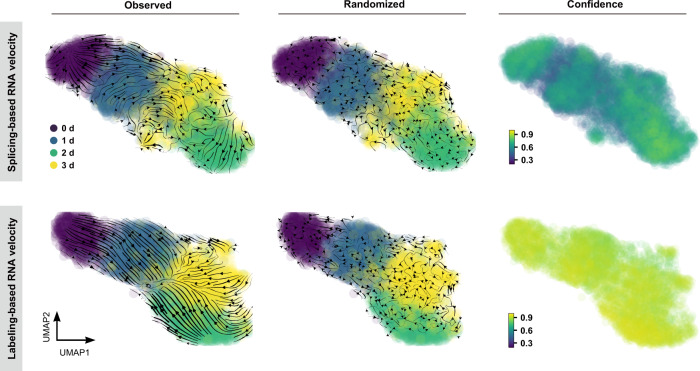
Metabolic labeling-based RNA velocity analysis of HCT116 cells in response to 5-AZA-CdR treatment. UMAP visualization of HCT116 cells treated with 5-AZA-CdR for 0/1/2/3 d characterized by conventional splicing-based (upper-left) or metabolic labeling-based (lower-left) RNA velocity analysis. Cells are color-coded by treatment time. The streamlines reveal the integration paths of local projections moving from the observed state to the extrapolated future state. The magnitude of RNA velocity is indicated by the streamline thickness. Randomized velocity controls (middle) were performed by first shuffling the velocity for genes of each cell and then randomly switching the sign of shuffled velocity values. The cell-wise velocity confidence (right) was measured by how well each velocity vector met the local neighborhood structure-defined geometric constraints.

We first examined whether the splicing-based model, which estimates RNA velocity by distinguishing unspliced mRNAs (intronic reads) from spliced mRNAs (exonic reads), can predict the transcriptional trajectory of single cells in response to 5-AZA-CdR treatment. As depicted in Fig. 5, poor 5-AZA-CdR treatment-dependent directionality was consistently observed in the splicing-based RNA velocity flow, lacking coherent transition of vectors. Since metabolic RNA labeling can capture rapid changes in RNA abundance and unambiguously quantify new RNAs via UMIs, we reason that single-cell paired measurements of newly transcribed and old mRNAs from Well-TEMP-seq can be utilized to determine metabolic labeling-based RNA velocity. As expected, the labeling-based model resulted in coherent velocity flows from untreated cells towards 5-AZA-CdR-treated cells in the low-dimensional embedding. Metabolic labeling-based RNA velocity of Well-TEMP-seq accurately recapitulated the temporal dynamics of gene expression of single cells upon 5-AZA-CdR treatment, including the first phase movement from 0 d to 1 d and 2 d and a second phase movement from 0 d to 1 d and 3 d. The randomized velocity control further confirmed the high specificity of the observed RNA velocity.

We also calculated the cell-wise confidence of velocity obtained from both models. Metabolic labeling-based RNA velocity exhibited higher confidence than splicing-based RNA velocity. Due to the sparsity of intronic reads from activity-induced genes and/or fast splicing kinetics, the capture of introns by the splicing-based model is highly biased (Supplementary Fig. 7). In contrast, the metabolic labeling-based strategy can label all new RNAs in a more uniform fashion (Supplementary Fig. 8). These results demonstrated that metabolic labeling-based RNA velocity outperformed splicing-based RNA velocity by accurately determining the observed and extrapolated cell states. The 5-AZA-CdR treatment-dependent state transition discovered by Well-TEMP-seq are difficult to be obtained by standard RNA velocity analysis. Therefore, Well-TEMP-seq supports the metabolic labeling-based and time-resolved RNA velocity analysis of dynamic gene expression in single cells.

## Discussion

Well-TEMP-seq combines metabolic RNA labeling by 4sU with our high-throughput Well-paired-seq scRNA-seq platform for massively parallel joint profiling of newly transcribed and pre-existing RNAs of the same cell. Well-TEMP-seq characterizes the temporal dynamics of single-cell gene expression of HCT116 cells exposed to a low dose of DNA demethylating 5-AZA-CdR, revealing the upregulation of TSGs, downregulation of oncogenes, and activation of antiviral interferon responsive immunity. Splicing-based RNA velocity is very straightforward to inform the future states of a cell by endogenous RNA splicing kinetics, but suffers from highly biased capture of intronic reads due to the sparsity of introns and fast splicing kinetics of many genes. In contrast, metabolic RNA labeling-based Well-TEMP-seq can directly count the new and old transcripts via UMIs to unbiasedly characterize the RNA kinetics for all detectable genes. Moreover, Well-TEMP-seq is capable of experimentally controlling the timing and length of metabolic RNA labeling periods, which makes it flexible to be applied in various biological processes.

Our Well-TEMP-seq is superior to other metabolic RNA labeling-based scRNA-seq methods such as scSLAM-seq, NASC-seq, scEU-seq, sci-fate, and scNT-seq (Supplementary Data 3). First, Well-TEMP-seq can handle up to 8 parallel samples at the same time with the throughput of thousands of single cells per sample. Second, Well-TEMP-seq and other UMI-based approaches require a lower sequencing depth than full-length transcript-based approaches. Third, Well-TEMP-seq costs <US$0.10 per cell for library preparation, which is even lower than other UMI-based approaches. It is noteworthy to mention that the key feature of Well-TEMP-seq is that it is built on the basis of Well-paired-seq with high accuracy and sensitivity. The size-exclusion and locally quasi-static hydrodynamic microwell-based microfluidic chip enable highly efficient loading of single cells (both live and fixed cells) into the microwells, removal of cell-free RNAs and cell clusters, and Poisson distribution-independent single cell/bead pairing. It overcomes the constraints of low cell/bead pairing efficiency and potential interference from cell-free RNAs in scNT-seq. Moreover, Well-TEMP-seq performs IAA chemistry on barcoded beads and thus avoids in situ chemical conversion and multiple centrifugation-induced cell loss as observed in sci-fate.

We anticipate that Well-TEMP-seq will be broadly applied to other complex and dynamic biological systems to characterize the temporal dynamics of single-cell gene expression. Most of the current metabolic RNA labeling experiments are performed with in vitro cell culture models. It is expected that future efforts will be committed to in vivo models by intravenous or intraperitoneal injection of exogenous nucleoside analogs to label newly transcribed RNAs in vivo. Development of new exogenous nucleoside analogs to confer other conversions (e.g., 6-thioguanosine to induce G-to-A conversion) in Well-TEMP-seq may enable independent recordings of different transcriptional processes in single cells to disentangle the complex interactions between different biological processes.

## Methods

### Mammalian cell culture

Human K562 cells (ATCC, CCL-243) and human colorectal cancer HCT116 cells (ATCC, CCL-247) were cultured in high glucose Dulbecco’s modified Eagle’s medium (DMEM) supplemented with 10% fetal bovine serum (Gibco, catalog no. 11965) and 1× penicillin-streptomycin (Gibco, catalog no. 15140122; 100 U/mL of penicillin, 100 µg/mL of streptomycin) and maintained at 37 °C with 5% CO_2_.

### Sample processing for Well-TEMP-seq

For K562 cells, cells were incubated with 200 μM 4sU for 2 h before cell harvest. For HCT116 cells, cells were treated with 300 nM 5-AZA-CdR for 0 day, 1 day, 2 days, or 3 days. Cells in each group were incubated with 200 μM 4sU for the last 2 h before cell harvest. The labeled HCT116 cells were trypsinized, spun down at 300 g for 5 min (4 °C), and washed once in 1× phosphate buffer saline (PBS). Cells (1 × 10^6^ − 5 × 10^6^ cells) were re-dispersed in 1 volume (100 μL) of ice-cold PBS (with 0.4 U/μL RNase inhibitor). The cell suspension was fixed with 9 volumes (900 μL) of ice-cold methanol (pre-chilled to −20 °C) for 10 min on ice in the dark. Methanol was added dropwise with a gentle vortex to avoid cell clumping. Fixed cells were centrifuged at 900 g for 3 min at 4 °C. Cell pellet was washed (not re-suspended) with ice-cold PBS (with 0.4 U/μL RNase inhibitor) and re-suspended in 100 μL enzyme blocking buffer (saturated ammonium sulfate solution with 50 mM EDTA, 0.8 U/μL RNase inhibitor, pH 5.2). The obtained cells were stored at −20 °C in the dark.

Cells were then re-suspended in PBS (with 0.05% bovine serum albumin) and subjected to cell loading, microbead loading, and cell lysis as described in the Well-paired-seq protocol. Briefly, single cells were loaded into the lower microwells by gravity, and cell clusters and cell-free RNAs were washed away by 1× PBS. After loading of microbeads, mineral oil with lysis aggregates (sakosyl) was introduced to cover the microwells. Sakosyl was quickly dissolved in the microwells containing PBS (water phase). Cells were then lysed and RNAs with poly(A) tails were captured by the oligo(dT) primers on microbeads. The recovered microbeads were pelleted and washed with 6× saline sodium citrate (SSC) and PBS (50 mM, pH 8.0) successively. For chemical conversion, microbeads were re-suspended in reaction buffer (50 mM PBS, 10 mM IAA, 10% DMSO, pH 8.0) and incubated at 37 °C for 1 h. Then 10 mM dithiolthreitol was added to stop the reaction. The microbeads were washed successively with 1× PBS and 1× RT buffer. After one-pot chemical conversion, the remaining library preparation steps were performed as described in Well-paired-seq. Briefly, for each group, microbeads (~10,000) were re-suspended in reverse transcription mix (1× Maxima reverse transcription buffer, 1 mM dNTPs, 1 U/μL RNase inhibitor, 2.5 μM template switching oligo (TSO: AAGCAGTGGTATCAACGCAGAGTGAATrGrGrG, Sangon Biotech), and 10 U/μL Maxima H Minus reverse transcriptase (catalog no. EP0751)). The reverse transcription reaction was proceeded at room temperature for 30 min, followed by incubation at 42 °C for 90 min. After Exonuclease I treatment (1 U/μL Exonuclease I, 37 °C, 45 min), microbeads were subjected to PCR reaction (1× KAPA HiFi hotstart readymix and 0.8 μM ISPCRoligo primer (AAGCAGTGGTATCAACGCAGAGT, Sangon Biotech)). Full-length cDNA was amplified by the following thermal cycling parameter (95 °C for 3 min; 4 cycles of (98 °C for 20 s, 65 °C for 45 s, and 72 °C for 3 min); 10–12 cycles of (98 °C for 20 s, 67 °C for 20 s and 72 °C for 3 min); 72 °C for 5 min and hold at 4 °C). The PCR product was purified twice using 0.6× VAHTS DNA Clean Beads (Vazyme Biotech, catalog no. N411-02) according to the manufacturer’s instructions. The 3′-end enriched sequencing library was prepared by TruePrep DNA Library Prep Kit V2 for Illumina (Vazyme Biotech, catalog no. TD503) according to the manufacturer’s instructions, except that P5 primer was replaced by a customized P5-TSO-hybrid primer (AATGATACGGCGACCACCGAGATCTACACGCCTGTCCGCGGAAGCAGTGGTATCAACGCAGAGT*A*C, Sangon Biotech). After quality control by Qsep-100, the library was sequenced on the Illumina Hiseq X Ten (paired-end, 150 bp). Read 1 primer was replaced by a customized Read 1 primer (GCCTGTCCGCGGAAGCAGTGGTATCAACGCAGAGTAC, Sangon Biotech).

### Read alignment and quantification

Paired-end sequencing reads of Well-TEMP-seq were processed as described in Well-paired-seq with some modifications. Briefly, reads were first processed using the Drop-seq pipeline (v2.3.0)^43^. For each pair of reads, the cell barcode (base 1–12) and UMI (base 13–20) in Read 1 were tagged to the mRNA read (Read 2). The obtained reads were then trimmed of sequencing adaptors and poly(A) sequences and aligned to the human reference genome assembly (GRCh38) using STAR v2.7.3a^44^. Different from the standard Drop-seq workflow, both exonic and intronic reads that mapped to predicted strands of annotated genes were retained for the downstream analysis. The pipeline of scNT-seq^20^ was adopted to further quantify metabolically labeled and unlabeled transcripts where uniquely mapped reads with a mapping score >10 and T-to-C substitutions with a base Phred quality score >27 were retained. Sites with T-to-C substitutions in the control group without 4sU labeling and IAA treatment were determined and excluded for T-to-C substitution identification in the experimental groups for correction of background mutation. Then a UMI/transcript was identified as labeled (newly transcribed) if there was at least one T-to-C substitution in any one of the mapped reads. For each gene, the total numbers of labeled and unlabeled mRNA were counted and assembled into matrices using the gene name as rows and the cell barcode as columns. Therefore, each cell was associated with two digital gene expression matrices (labeled and unlabeled transcripts).

### Estimation of the portion of newly transcribed transcripts

To address the insufficiency of metabolic RNA labeling, we adopted a statistical model to approximate the real distribution of T-to-C substitution in single cells and estimate the real mutation rate. There are two kinds of T-to-C substitution in a single cell. One is endogenous and the other is induced by exogenous labeling. They behave according to different distributions. A previous study showed that regardless of the kind of substitution, the labeling rates for each gene are quite similar in the same cell^20^. Therefore, here we used a binomial mixture model:

1 $$f\left(\theta,p,q\right)=\theta {{{{{\rm{Binom}}}}}}\left({y}_{i}{{{{{\rm{;}}}}}}p,{n}_{i}\right)+\left(1-\theta \right){{{{{\rm{Binom}}}}}}\left({y}_{i}{{{{{\rm{;}}}}}}q,{n}_{i}\right)$$

2 $${{{{{\rm{Binom}}}}}}\left({y}_{i}{{{{{\rm{;}}}}}}p,{n}_{i}\right)=\left(\begin{array}{c}{n}_{i}\\ {y}_{i}\end{array}\right) \, {{\cdot }} \, {p}^{{y}_{i}}{\left(1-p\right)}^{{n}_{i}-{y}_{i}}$$

3 $${{{{{\rm{Binom}}}}}}\left({y}_{i}{{{{{\rm{;}}}}}}q,{n}_{i}\right)=\left(\begin{array}{c}{n}_{i}\\ {y}_{i}\end{array}\right) \, {{\cdot }} \, {q}^{{y}_{i}}{\left(1-q\right)}^{{n}_{i}-{y}_{i}}$$

The sufficient data for this model are the number of uridine nucleotides (${n}_{i}$) observed in transcript *i* and the corresponding mutations (${y}_{i}$) for each transcript *i*. $\theta$ is the portion of newly transcribed mRNA among all mRNAs in each experiment, then $p$ is the mutation rate in labeled mRNAs and $q$ is the mutation rate in unlabeled mRNAs. To avoid sequence error, we generated a consensus sequence for each transcript by gathering reads with the same UMI index and picking the most frequent variant at each site. UMIs with fewer than 2 reads were filtered. 100000 consensus sequences were randomly selected to compute p and q in the above model for each time point. The built likelihood function was maximized by the Nelder-Mead algorithm and repeated 100 times to obtain the maximum function value. The corresponding p and q were chosen as final parameters (achieved by nloptr in R v4.0.0)^45^.

The obtained 4 sets of p and q (4 time points) were used for calculating ${\theta }_{{gene}}$, the level of new transcript for each gene at each time point, according to the above statistical model. Since the detection rate *α* of most genes are highly similar in one cell, the mean detection rate *α* is obtained by dividing the number of all observed labeled transcripts by the number of all estimated new transcripts for each cell and can be calculated as

4 $${\alpha }_{{cell}}=\frac{{L}_{{cell}}}{{\sum \theta }_{{gene}}({L}_{{gene}}+{U}_{{gene}})}$$

After calculating ${\alpha }_{{cell}}$, the estimated new RNA level for each gene in each cell can be calculated as

5 $${N}_{{gene}}={{\min }}\left(\frac{{L}_{{gene}}}{{\alpha }_{{cell}}},\left({L}_{{gene}}+{U}_{{gene}}\right)\right)$$

where ${L}_{{gene}}$ is the number of labeled RNA of a gene in that cell and ${U}_{{gene}}$ is the number of unlabeled RNA of the same gene in that cell. The computed new and old transcripts were used for all downstream single-cell analyses, including differential gene expression analysis, SCENIC-based single-cell regulon activity analysis, and RNA velocity analysis.

### Identification of differentially expressed genes (DEGs)

Differential gene expression analysis of RNAs between different time points of 5-AZA-CdR treatment (1/2/3 d) and control (0 d) was carried out with the FindMarkers function in Seurat package using the default Wilcoxon rank-sum test. Genes with an absolute fold change >1.5 and an adjusted *P* value <0.05 (Bonferroni corrected) in expression between treatment groups (at least one group) and control group were considered as differentially expressed genes. Mitochondrial genes and ribosomal protein-coding genes were excluded for downstream analysis. The identified DEGs were divided into two groups (i.e., the up-regulated group and the down-regulated group).

### Analysis of single-cell regulon activity

Regulon activities of TFs were quantified by linking *cis*-regulatory sequences to single-cell gene expression as demonstrated by the recently proposed single-cell regulatory network inference and clustering (SCENIC) method^27,28^. Regulon modules were identified by inferring the coexpression of TFs and sets of target genes containing TF-binding motifs in their promotors. First, the gene expression matrix (genes expressed in fewer than 20 cells were removed) was separated into two parts (i.e., new transcripts and old transcripts) and saved in RDS format. The RDS files were transformed into loom format and provided as inputs for SCENIC analysis. Therefore, specific regulon modules associated with either new or old transcriptomes from the same cell could be identified separately. Coexpression modules were inferred by GRNBoost (implemented in pySCENIC v0.11.2) and the weight between TFs and their target genes was quantified. Target genes that failed to show a positive correlation (>0.03) in each TF module were removed. Second, cisTarget was adopted to perform *cis*-regulatory motif enrichment of target TF motifs in the 10-kb window around genes’ promoters and to identify putatively direct targets. Indirect targets without significant motif enrichment of the correct upstream regulator were discarded. Third, the activities of the obtained TF regulon modules in each cell were calculated by the AUCell algorithm implemented in pySCENIC. For each TF, the mean AUC value of all cells belonging to the same group was computed and scaled by the scale function in R. The TF regulon activities of different groups and clustering were visualized by heat map using the pheatmap package (v1.0.12) in R. Two-side Wilcoxon rank-sum test was performed to quantify the significance of difference for TF activity. Regulons with an absolute fold change of regulon activity > 1.5 and an adjusted *P* value <0.05 (Bonferroni corrected) between treated groups (at least one group) and untreated group (i.e., 1 d vs. 0 d, 2 d vs. 0 d, 3 d vs. 0 d) were considered as 5-AZA-CdR treatment responsive regulons.

### Splicing-based RNA velocity analysis

Spliced and unspliced counts are required for standard RNA velocity (splicing-based RNA velocity). First, we adopted the Drop-seq pipeline to generate the aligned bam file^46^. Second, the dropEst function in dropEst pipeline was applied for demultiplexing reads to separate spliced and unspliced reads. The parameter was set as ‘-M -V -b -f -L eiEIBA’ and the GRCh38 genome annotations were used. Dynamo, the computational framework for both standard and metabolic labeling-based RNA velocity analysis, was then used for RNA velocity analysis. The RNA dynamics and velocity information was calculated with the model of “auto”. Ultimately, we mapped the high-dimensional velocity vectors to 2-dimensional UMAP space and realized the visualization by streamline plot, randomized streamline plot, and phase diagrams with default settings.

### Labeling-based RNA velocity

For metabolic labeling-based RNA velocity analysis, the new transcript and total transcript counts are required. First, the scNT pipeline^20^ was adopted to separate and count new transcripts and old transcripts. After correcting the transcriptome profiles of new RNAs, the data for new transcripts and total transcripts were loaded into dynamo. The model was set as “auto” and the option of “NTR_vel” was defined as “True”. Similar to splicing-based RNA velocity analysis, the streamline plot, randomized streamline plot, and phase diagrams were visualized with default settings.

### Western blotting

HCT116 cells were treated with 300 nM 5-AZA-CdR for different durations (0 d, 1 d, 2 d, 3 d). Cells were lysed in RIPA buffer (Beyotime, catalog no. P0013E) supplemented with protease inhibitor (Beyotime, catalog no. ST506) for protein extraction. The equal weight of denatured proteins (20 μg) were separated by SDS-PAGE and then transferred onto a PVDF membrane (Millipore). The membrane was blocked in 5% non-fat milk dissolved in Tris-buffered saline with Tween-20 (TBST) for 1 h and then washed by TBST three times. Subsequently, the membrane was incubated with rabbit anti-human primary antibodies against STAT1 (phospho Y701) (Abcam, catalog no. ab109457, 1:1000 dilution) or α-tubulin (Abbkine, catalog no. ABP52655, 1:1000 dilution) overnight at 4°C and horseradish peroxidase-labeled secondary antibody (Abcam, catalog no. ab97080, 1:10000 dilution) at room temperature for 1 h. The signals of protein were developed by ECL Kit (Beyotime, catalog no. P0018S).

### Statistics & reproducibility

No statistical method was used to predetermine sample size. No data were excluded from the analyses. Two biologically independent replicates were included for K562 Well-TEMP-seq. The technique was also tested and validated in other cell lines. For HCT116 Well-TEMP-seq, no replication was performed for reasons of scale and cost. The order of samples are randomized during drug treatment, and during sample processing in Well-TEMP-seq. For estimation of the fraction of new transcripts, 100,000 consensus sequences were randomly selected to compute p and q in the binomial mixture model for each time point. Investigators were blinded to group allocation during data collection (sequencing) and analysis.

### Reporting summary

Further information on research design is available in the Nature Portfolio Reporting Summary linked to this article.

## Supplementary information

Supplementary information

Description of Additional Supplementary Files

Supplementary Data 1

Supplementary Data 2

Supplementary Data 3

Reporting Summary

## Data Availability

All sequencing data can be downloaded from Gene Expression Omnibus (GEO) with accession code of “GSE194357”. The human reference genome (GRCh38) used in this study can be downloaded from https://asia.ensembl.org/index.html. Source data are provided with this paper.

## Source data

Source data

## Code availability

The source code for the analysis of Well-TEMP-seq data is available on GitHub (https://github.com/songjiajia2018/Well-TEMP-Seq).
