# Supplementary material for: Well-TEMP-seq as a microwell-based strategy for massively parallel profiling of single-cell temporal RNA dynamics
Source: Nat Commun. 2023 Mar 7;14:1272. doi: 10.1038/s41467-023-36902-5 (PMC9992361; doi:10.1038/s41467-023-36902-5)
Supplement: Supplementary file 1 — Supplementary information [file 41467_2023_36902_MOESM1_ESM.pdf]

# Supplementary Information

# Well-TEMP-seq as a microwell-based strategy for massively parallel profiling of the single-cell temporal RNA dynamics

Lin et al.

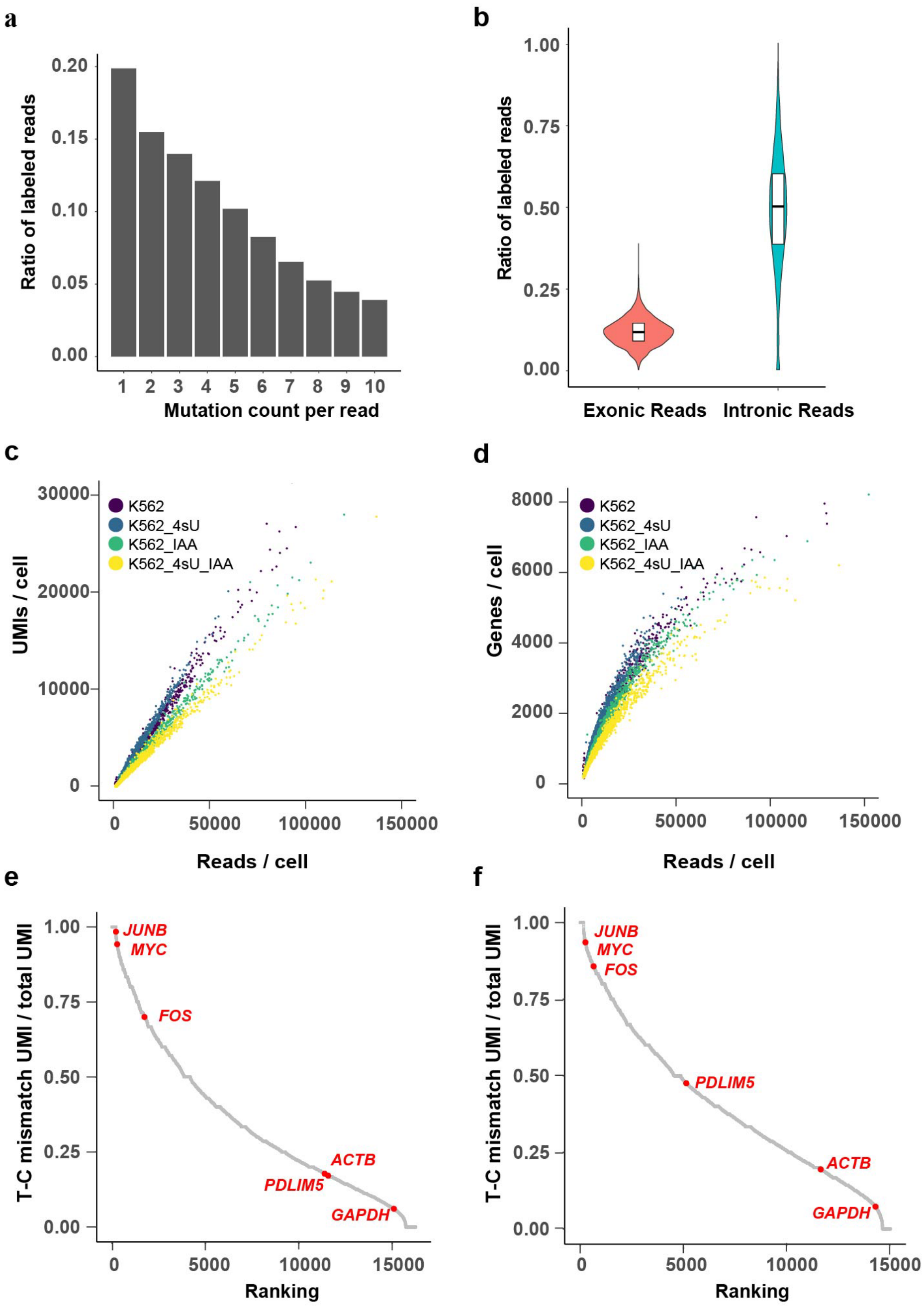


**Supplementary Fig. 1 | Performance and quality control analyses of Well-TEMP-seq.**

(a) Bar plot showing the distribution of T-to-C mutation counts across labeled reads of 4sU-labeled and IAA-treated K562 cells. (b) Violin plot showing the ratio of 4sU-labeled reads per cell of 4sU-labeled and IAA-treated K562 cells, split out by the subsets that map to exons versus introns (n = 5000 cells for each group. Boxplots include centerline, median; box limits, upper and lower quartiles). (c) Scatter plot showing the number of UMI detected per cell as a function of aligned reads per cell in

different groups. (d) Scatter plot showing the number of gene detected per cell as a function of aligned reads per cell in different groups. Quantitative information on the recovery rate and respective loss of transcripts (UMIs) based on the observed complexities of the obtained sequencing libraries are provided in Supplementary Data 1. (e) Global analysis of the average fraction of T-C mismatch UMIs per cell for all detected genes in 4sU-labeled and IAA-treated K562 cells. (f) Global analysis of the average fraction of T-C mismatch UMIs per cell for all detected genes in 4sU-labeled and IAA-treated HCT116 cells. Source data are provided as a Source Data file.

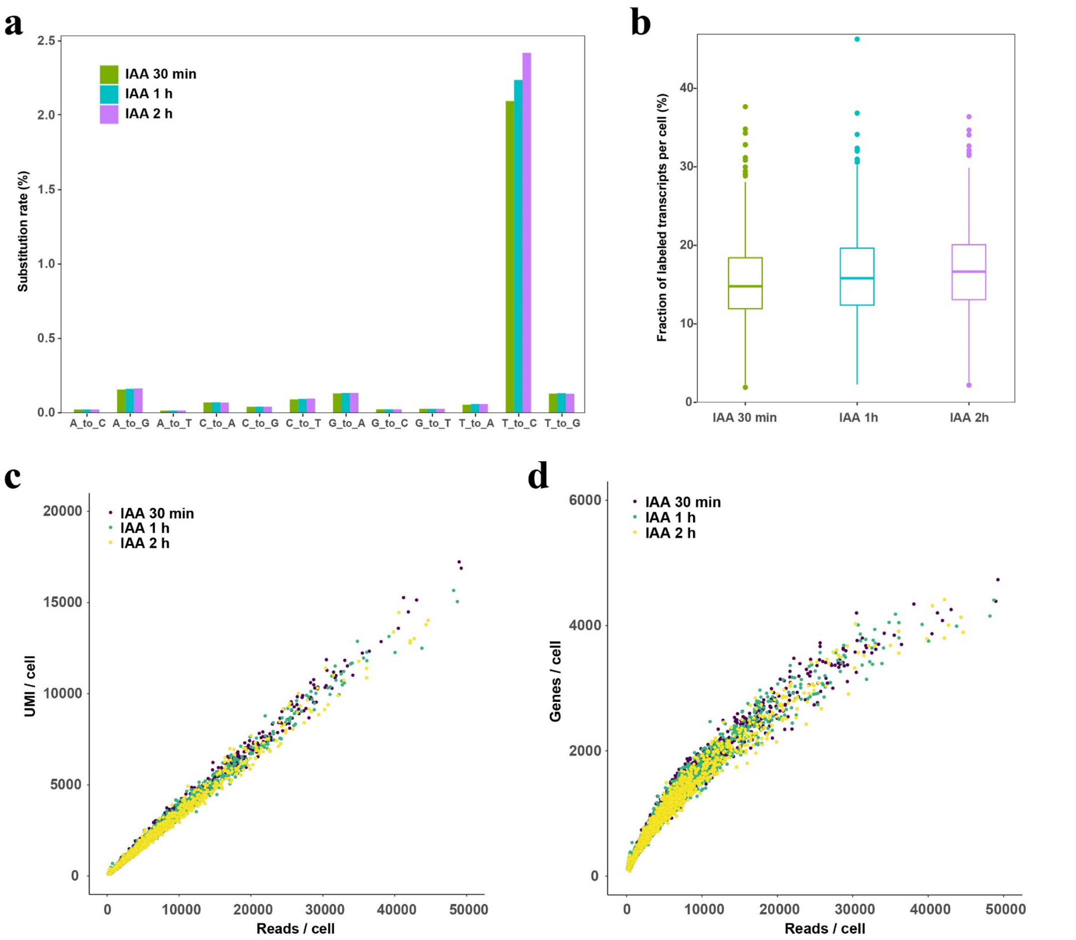


**Supplementary Fig. 2 | Optimization of the IAA chemical treatment duration in Well-TEMP-seq.**

(a) Bar plot of nucleotide substitution rates in 4sU-labeled K562 cells treated with IAA for various durations (30 min, 1 h, and 2 h). (b) Box plot of the fraction of labeled transcripts per cell in 4sU-labeled K562 cells treated with IAA for various durations (30 min, 1 h, and 2 h; n = 845 (IAA 30 min), 875 (IAA 1 h), 850 (IAA 2 h) cells. Boxplots include centerline, median; box limits, upper and lower quartiles; and whiskers are highest and lowest values no >1.5× interquartile range). (c) Scatter plot showing the number of UMIs detected per cell as a function of aligned reads per cell in different groups with various IAA treatment durations (30 min, 1 h, and 2 h). (d) Scatter plot showing the number of genes detected per cell as a function of aligned reads per cell in different groups with various IAA treatment durations (30 min, 1 h, and 2 h). The T-to-C conversion rate and the fraction of labeled transcripts both increased as the treatment duration increased while the library complexity decreased slightly when the treatment

duration increased. To strike a balance between the labeling efficiency and the library complexity, we selected the IAA conversion duration of 1 h for Well-TEMP-seq. Source data are provided as a Source Data file.

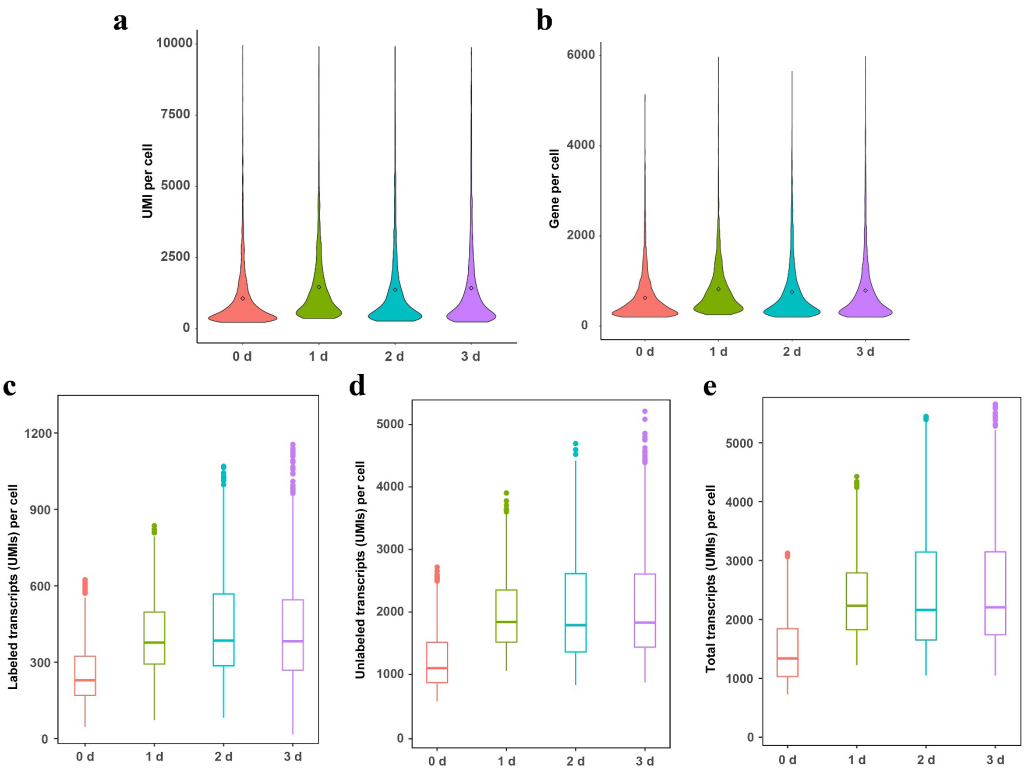


**Supplementary Fig. 3 | Transcripts and genes covered in HCT116 cells treated with 5-AZA-CdR.**

(a) Violin plots of the number of detected UMIs per cell of different groups treated with 5-AZA-CdR. (b) Violin plots of the number of detected genes per cell of different groups treated with 5-AZA-CdR. (c) Box plots of the absolute number of labeled UMIs per cell in different groups treated with 5-AZA-CdR. (d) Box plots of the absolute number of unlabeled UMIs per cell in different groups treated with 5-AZA-CdR. (e) Box plots of the absolute number of total UMIs per cell in different groups treated with 5-AZA-CdR. In **c-e**, n = 872 (0 d), 867 (1 d), 884 (2 d), 832 (3 d) cells and boxplots include centerline, median; box limits, upper and lower quartiles; and whiskers are highest and lowest values no >1.5× interquartile range. Source data are provided as a Source Data file.

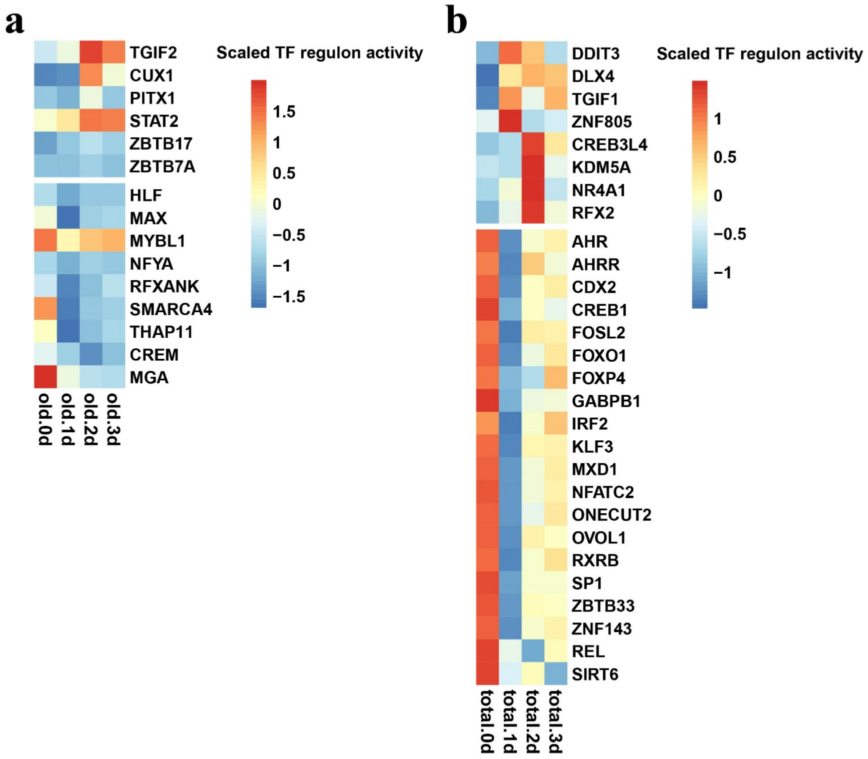


**Supplementary Fig. 4 | Old RNA-based and total RNA-based SCENIC analysis.**

(a) Heat map showing average regulon activity of 5-AZA-CdR treatment-dependent regulons in HCT116 cells inferred from old RNAs. (b) Heat map showing average regulon activity of 5-AZA-CdR treatment-dependent regulons in HCT116 cells inferred from total RNAs. Source data are provided as a Source Data file.

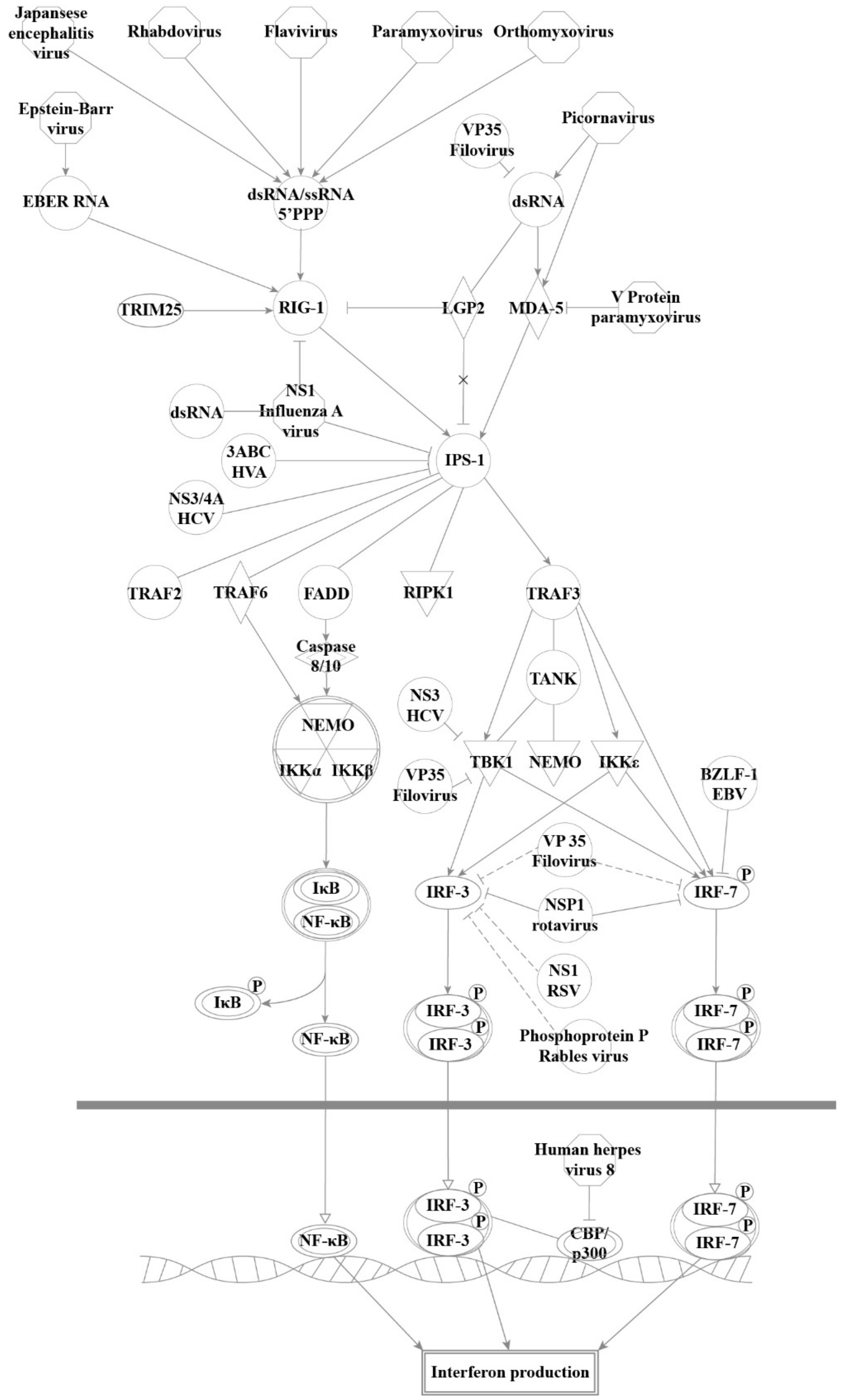


**Supplementary Fig. 5 | Antiviral innate immunity pathway.**

Diagram of the canonical pathway entitled "role of RIG1-like receptors in antiviral innate immunity" from the Ingenuity Pathway Analysis software[1].

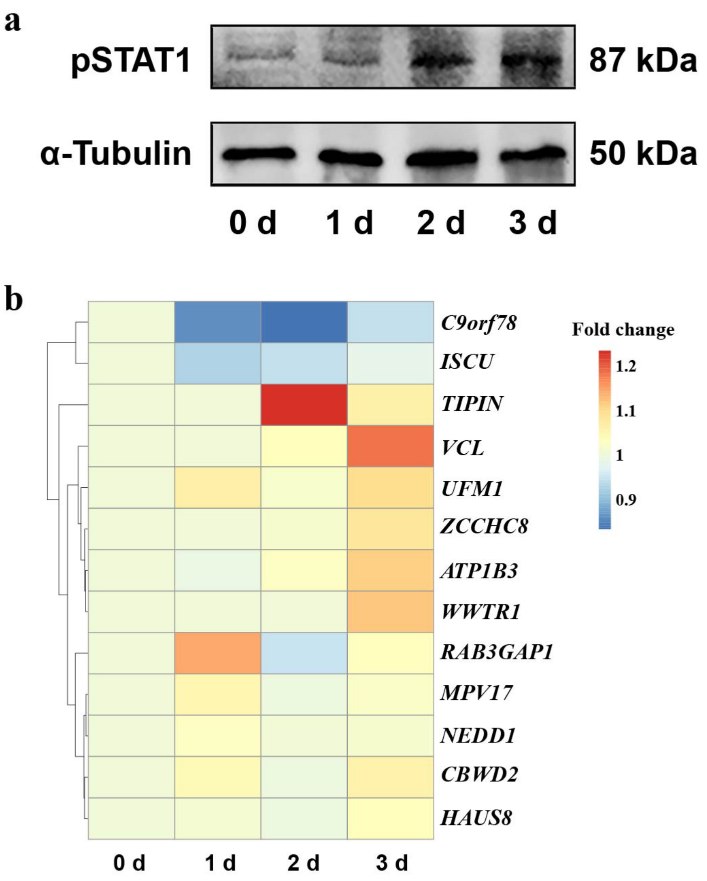


**Supplementary Fig. 6 | Activation of STAT1 and downstream target genes.**

(a) Immunoblot of cellular extracts in response to 5-AZA-CdR treatment. HCT116 cells were treated with 300 nM 5-AZA-CdR for different durations (0/1/2/3 d) and then the cellular proteins were extracted for western blotting. Phospho-Stat1 (Tyr701) monoclonal antibody was used. Two biological replicates were performed to confirm the results. (b) Clustered heat map showing the new RNA levels of STAT1 target genes in response to 5-AZA-CdR treatment. The target genes were identified from single-cell new transcriptomes by SCENIC. Source data are provided as a Source Data file.

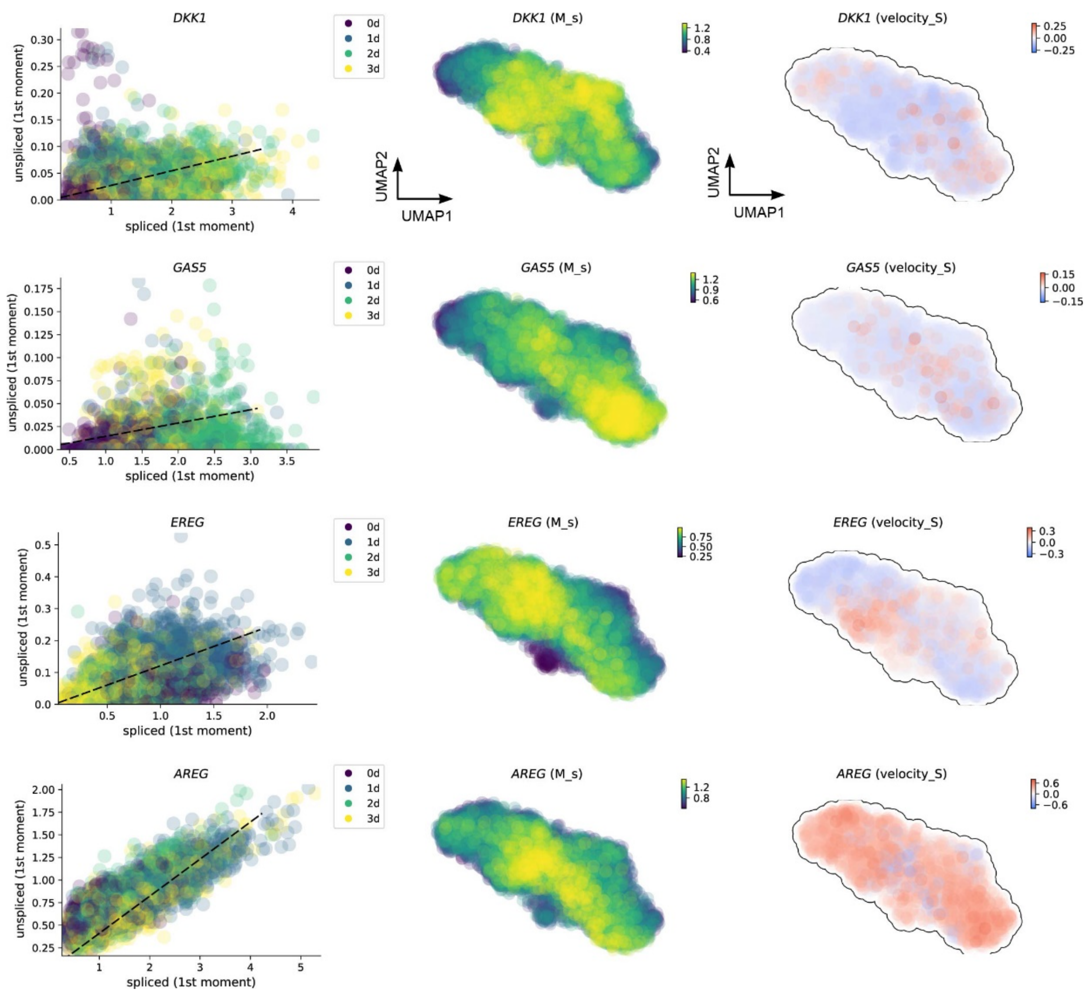


**Supplementary Fig. 7 | Performance of splicing-based RNA velocity analysis.**

Phase portraits (left), UMAP plots colored by smoothed spliced RNA level based on local averaging (middle), and RNA velocity values (right) of four representative 5-AZA-CdR treatment responsive genes (*DKK1*, *GAS5*, *EREG*, and *AREG*).

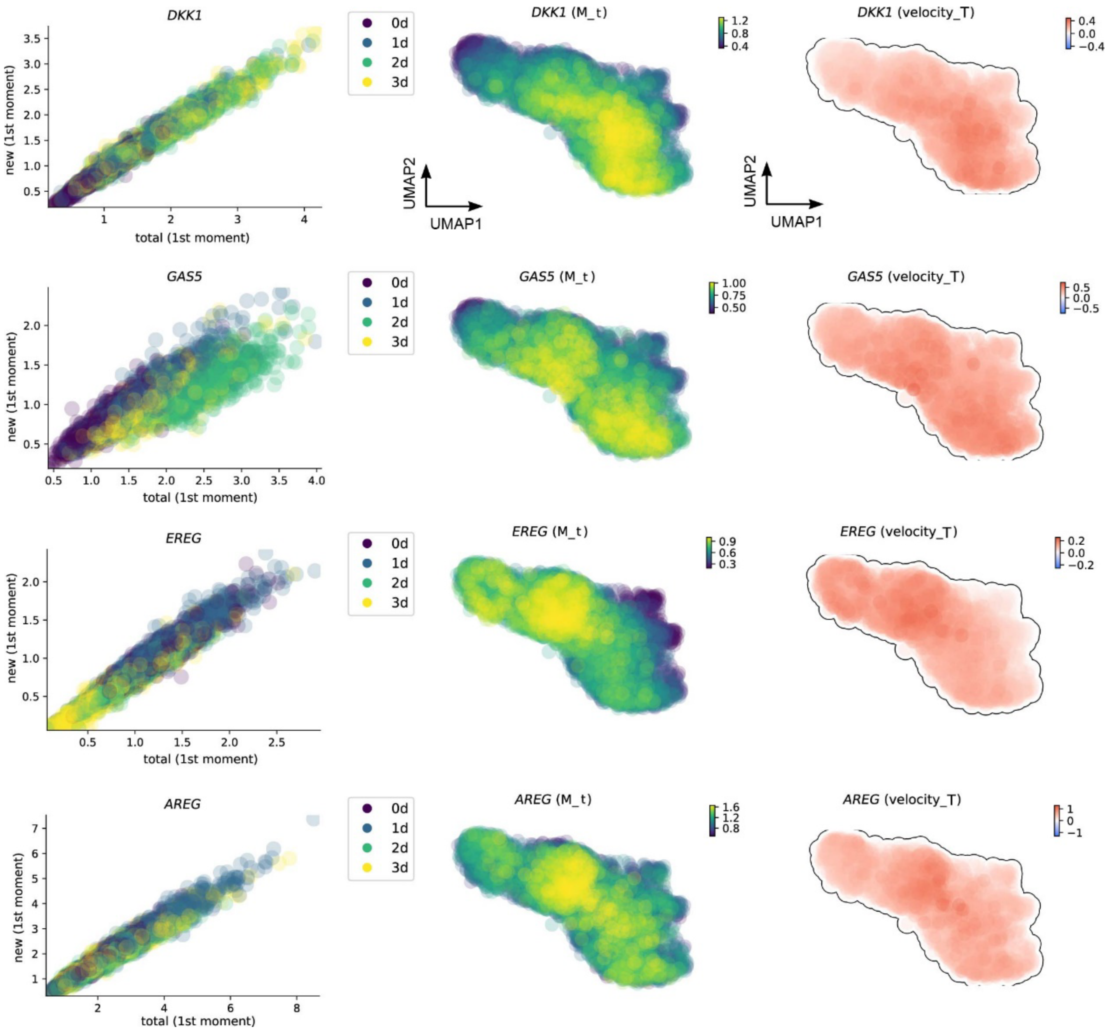


**Supplementary Fig. 8 | Performance of metabolic labeling-based RNA velocity analysis.**

Phase portraits (left), UMAP plots colored by smoothed total RNA level based on local averaging (middle), and RNA velocity values (right) of four representative 5-AZA-CdR treatment responsive genes (*DKK1*, *GAS5*, *EREG*, and *AREG*).

## Supplementary references

[1] Roulois, D., Loo Yau, H., Singhania, R., Wang, Y., Danesh, A., Shen, Shu Y., Han, H., Liang, G., Jones, Peter A., Pugh, Trevor J., O'Brien, C. and De Carvalho, Daniel D. DNA-Demethylating Agents Target Colorectal Cancer Cells by Inducing Viral Mimicry by Endogenous Transcripts. *Cell* **162**, 961-973 (2015).
