# Supplementary material for: Well-TEMP-seq as a microwell-based strategy for massively parallel profiling of single-cell temporal RNA dynamics
Source: Nat Commun. 2023 Mar 7;14:1272. doi: 10.1038/s41467-023-36902-5 (PMC9992361; doi:10.1038/s41467-023-36902-5)
Supplement: Supplementary file 2 — Description of Additional Supplementary Files [file 41467_2023_36902_MOESM2_ESM.pdf]

Description of Additional Supplementary Files

Title: Supplementary Data 1
Description: Quantitative information of the detected UMIs and genes per cell in the K562 cell

Title: Supplementary Data 2
Description: The mean UMIs and genes per cell and respective standard deviations of different groups

Title: Supplementary Data 3
Description: Comparison of different single-cell metabolic labeling-based RNA-sequencing
