# Supplementary material for: Well-TEMP-seq as a microwell-based strategy for massively parallel profiling of single-cell temporal RNA dynamics
Source: Nat Commun. 2023 Mar 7;14:1272. doi: 10.1038/s41467-023-36902-5 (PMC9992361; doi:10.1038/s41467-023-36902-5)
Supplement: Supplementary file 6 — Reporting Summary [file 41467_2023_36902_MOESM6_ESM.pdf]

nature portfolio

Corresponding author(s): Chaoyong Yang, Jia Song

Last updated by author(s): Feb 9, 2023

Reporting Summary

Nature Portfolio wishes to improve the reproducibility of the work that we publish. This form provides structure for consistency and transparency in reporting. For further information on Nature Portfolio policies, see our Editorial Policies and the Editorial Policy Checklist.

Statistics

For all statistical analyses, confirm that the following items are present in the figure legend, table legend, main text, or Methods section.

n/a	Confirmed	
☐	☒	The exact sample size (*n*) for each experimental group/condition, given as a discrete number and unit of measurement
☐	☒	A statement on whether measurements were taken from distinct samples or whether the same sample was measured repeatedly
☐	☒	The statistical test(s) used AND whether they are one- or two-sided<br>*Only common tests should be described solely by name; describe more complex techniques in the Methods section.*
☒	☐	A description of all covariates tested
☐	☒	A description of any assumptions or corrections, such as tests of normality and adjustment for multiple comparisons
☐	☒	A full description of the statistical parameters including central tendency (e.g. means) or other basic estimates (e.g. regression coefficient) AND variation (e.g. standard deviation) or associated estimates of uncertainty (e.g. confidence intervals)
☐	☒	For null hypothesis testing, the test statistic (e.g. *F*, *t*, *r*) with confidence intervals, effect sizes, degrees of freedom and *P* value noted<br>*Give P values as exact values whenever suitable.*
☒	☐	For Bayesian analysis, information on the choice of priors and Markov chain Monte Carlo settings
☒	☐	For hierarchical and complex designs, identification of the appropriate level for tests and full reporting of outcomes
☒	☐	Estimates of effect sizes (e.g. Cohen's *d*, Pearson's *r*), indicating how they were calculated

Our web collection on statistics for biologists contains articles on many of the points above.

Software and code

Policy information about availability of computer code

Data collection	No software was used for data collection.
Data analysis	Drop-seq pipeline (v2.3.0), STAR (v2.7.3a), nloptr (v4.0.0), pheatmap (v1.0.12), pySCENIC (v0.11.2), dropEst (v0.8.5), dynamo (v1.1.0), scNT-seq pipeline (v1.0.0, https://github.com/wulabupenn/scNT-seq).The source code for the analysis of Well-TEMP-seq data is available on GitHub (https://github.com/songjiajia2018/Well-TEMP-Seq).

For manuscripts utilizing custom algorithms or software that are central to the research but not yet described in published literature, software must be made available to editors and reviewers. We strongly encourage code deposition in a community repository (e.g. GitHub). See the Nature Portfolio guidelines for submitting code & software for further information.

Data

Policy information about availability of data

All manuscripts must include a data availability statement. This statement should provide the following information, where applicable:

- Accession codes, unique identifiers, or web links for publicly available datasets
- A description of any restrictions on data availability
- For clinical datasets or third party data, please ensure that the statement adheres to our policy

All sequencing data in this study can be downloaded from the NCBI Gene Expression Omnibus (GEO) with accession code of GSE194357.The human reference genome (GRCh38) used in this study can be download from https://asia.ensembl.org/index.html.

Human research participants

Policy information about studies involving human research participants and Sex and Gender in Research.

Reporting on sex and gender	n/a
Population characteristics	n/a
Recruitment	n/a
Ethics oversight	n/a

Note that full information on the approval of the study protocol must also be provided in the manuscript.

Field-specific reporting

Please select the one below that is the best fit for your research. If you are not sure, read the appropriate sections before making your selection.

☒ Life sciences ☐ Behavioural & social sciences ☐ Ecological, evolutionary & environmental sciences

For a reference copy of the document with all sections, see nature.com/documents/nr-reporting-summary-flat.pdf

Life sciences study design

All studies must disclose on these points even when the disclosure is negative.

Sample size	No explicit calculations were performed to determine sample size. For the sample size and the number of repetitions for each experiment, we referred to the design in the most published studies.
Data exclusions	Data were not excluded.
Replication	Two biologically independent replicates were included for K562 Well-TEMP-seq. The technique was also tested and validated in other cell lines. For HCT116 Well-TEMP-seq, no replication was performed for reasons of scale and cost.
Randomization	The order of samples are randomized during drug treatment, and during sample processing in Well-TEMP-seq. Samples have been labeled with drug treatment duration information before sequencing and were used for estimation of the fraction of new transcripts. For estimation of the fraction of new transcripts, 100000 consensus sequences were randomly selected to compute p and q in the binomial mixture model for each time point.
Blinding	Investigators were blinded to group allocation during data collection (sequencing) and analysis.

Reporting for specific materials, systems and methods

We require information from authors about some types of materials, experimental systems and methods used in many studies. Here, indicate whether each material, system or method listed is relevant to your study. If you are not sure if a list item applies to your research, read the appropriate section before selecting a response.

Materials & experimental systems

n/a	Involved in the study	
☐	☒	Antibodies
☐	☒	Eukaryotic cell lines
☒	☐	Palaeontology and archaeology
☒	☐	Animals and other organisms
☒	☐	Clinical data
☒	☐	Dual use research of concern

Methods

n/a	Involved in the study	
☒	☐	ChIP-seq
☒	☐	Flow cytometry
☒	☐	MRI-based neuroimaging

Antibodies

Antibodies used	Anti-STAT1 (phospho Y701), 1:1000 dilution (Abcam: ab109457, rabbit)<br>Anti-α-Tubulin, 1:1000 dilutioin (Abbkine: ABP52655, rabbit)<br>Anti-Rabbit IgG H&L (HRP) preadsorbed, 1:10000 dilution (Abcam: ab97080, goat)

Validation	Anti-STAT1 (phospho Y701), 1:1000 dilution (Abcam: ab109457, rabbit). Reacts with: Human. Suitable for: WB, Dot blot. Citations from manufacturer are listed at https://www.abcam.cn/stat1-phospho-y701-antibody-epr3147-ab109457.html.<br>Anti-α-Tubulin, 1:1000 dilution (Abbkine: ABP52655, rabbit).Reacts with: Human, Mouse, Rat. Suitable for: WB, ELISA, IF, IHC-P. Citations from manufacturer are listed at https://www.abbkine.com/product/tubulin-α-polyclonal-antibody-abp52655/<br>Anti-Rabbit IgG H&L (HRP) preadsorbed, 1:10000 dilution (Abcam: ab97080, goat). Suitable for: ICC, IHC-P, ELISA, WB. Citations from manufacturer are listed at https://www.abcam.com/goat-rabbit-igg-hl-hrp-preadsorbed-ab97080.html.

Eukaryotic cell lines

Policy information about cell lines and Sex and Gender in Research

Cell line source(s)	K562 and HCT116 cells were from ATCC.
Authentication	None of the cell lines were authenticated.
Mycoplasma contamination	Not tested.
Commonly misidentified lines (See ICLAC register)	No commonly misidentified cell lines were used.
